# Implications of Age on Social Media Utilization in Health Care Practice Development: Cross-sectional Survey Study

**DOI:** 10.2196/27528

**Published:** 2021-07-15

**Authors:** Harrison Marsh, Mhd Hasan Almekdash, Stephen Rossettie, Albin John, Kassie Pelham, Brent Magers

**Affiliations:** 1 Texas Tech University Health Sciences Center Lubbock, TX United States; 2 Texas Tech Physicians Group Lubbock, TX United States

**Keywords:** social media, health care, age, medical practice development, patient acquisition, health care delivery, patient education, target patient population

## Abstract

**Background:**

Medical practices, which are businesses through which one or more physicians treat patients, have likely not yet taken full advantage of the reach of social media. This study analyzed data collected using an anonymous survey to assess the potential utilization of large, established social media platforms in health care. The survey collected data from a diverse population of health care professional students, faculty, and physicians affiliated with the Texas Tech University Health Sciences Center (TTUHSC). This study provides significant, actionable data to more efficiently implement a social media strategy focused on age to help developing private practices and outpatient clinics from the perspective of those with experience in the field of medicine.

**Objective:**

This cross-sectional, exploratory, descriptive study aims to explore the most effective strategies to use social media based on patient age to bring further success to a medical practice.

**Methods:**

Data were gathered from an anonymous, peer-validated Qualtrics survey created by the corresponding authors based on the recommendations from a panel of experts including executive leadership at TTUHSC. The survey used a variety of question styles to measure differences between social media platforms, including frequency of use, current and future implications in medicine, and comfort in a health care setting. The sample population included students, interns, faculty, and physicians affiliated with the TTUHSC located throughout West Texas.

**Results:**

The anonymous survey included 673 individuals from several different age groups predetermined at the beginning of the study. There were 154 respondents aged between 18 and 25 years, 171 aged between 26 and 35 years, 133 aged between 36 and 45 years, 104 aged between 46 and 55 years, and 111 aged between 56 and 89 years. The sample population also has a variety of educational achievements. The respondents were grouped based on the highest level of education attained, and this included 23.5% (n=158) of respondents who earned a high school diploma, 42% (n=283) who earned a bachelor’s degree, 17.1% (n=115) who earned a master’s degree, and 17.4% (n=117) who earned a doctorate degree.

**Conclusions:**

As social media continues to gain momentum, efficient utilization of the available platforms can help medical practices achieve larger patient populations and deliver more personalized care. However, privacy and security concerns should be considered while using social media in health care settings. Although this study demonstrated overwhelming interest in using social media in the medical field across all age groups, adoption willingness appears to be higher in younger respondents than in older respondents. Facebook was the most widely accepted social media platform in health care settings among all age groups. Nonetheless, other social media platforms could potentially be used more effectively depending on the age range of the targeted patient population.

## Introduction

### Background

Nearly half of the world’s population now uses social media (approximately 3.5 billion). According to the Pew Research Center, in 2005, only 5% of American adults used at least one of these platforms. However, by 2011, that share had risen to 50% of all Americans, and in 2019, 72% of Americans reported using one of the social media platforms [1]. Considering the rapid growth and vast use, there is no doubt that social media can be used to bring further success to the medical field. The difficulty is how to best optimize this tool among varying patient populations [2,3]. Several medical institutions and private practices now broadcast recurring podcasts, YouTube videos, and other forms of social media [4]. For example, in 2019, the Texas Tech Physicians implemented paid Facebook advertising targeting current and prospective patients in West Texas to attract interest to the physicians and improve general health care screening in the area. This initiative drove a growth of over 500% in their web-based following. However, two obstacles prevent the consistent optimization of these tools. First, the direct and indirect benefits of social media are yet to be measured. Second, the best methods to capitalize on social media for new or growing medical practices are yet to be completely explored. Having seen the success of social media use in already-established groups such as Texas Tech Physicians, it is very likely that physicians interested in attempting to open a new private medical practice would also benefit from social media implementation to establish a good reputation, especially during the early stages of the practice [5]. The information and conclusions gathered from this research could greatly benefit anyone trying to improve the patient acquisition, patient satisfaction, or overall health care delivery of a medical practice [6].

Several direct benefits of using social media in health care have been identified, including increased interactions with patients, increased information accessibility, further tailored information, improved peer, social, and emotional support, increased public health surveillance, and greater potential to influence health policy [7,8]. With the rapid development and improvement of social media platforms, these benefits are only the beginning of the potential improvements that could be made through social media utilization [9]. The questions that remain are as follows: what forms of social media would lead to the greatest success, what percentage of patients from different backgrounds would social media utilization likely benefit, and what indirect benefits could arise from proper utilization of these platforms.

### Objectives

Some of the challenges of social media utilization in medical practice have already been identified as quality, reliability, confidentiality, and privacy concerns [7,10,11]. However, social media has made improvements in these areas of concern such that the current benefits may outweigh the risks [12]. Although these apprehensions with social media utilization should still be addressed in further studies, this study will focus more on the opportunities of efficient social media use in the health care setting by focusing on differences in social media utilization and preference based on age.

## Methods

### Study Design and Sample

This was a cross-sectional survey design, exploratory, and descriptive investigation. The Institutional Review Boards at Lubbock and Odessa approved this protocol and waived the requirement for informed consent.

The possible benefits of social media utilization were measured through data gathered from an anonymous survey evaluating different perspectives of faculty, staff, and students of all backgrounds, ages, and education levels affiliated with the Texas Tech University Health Sciences Center (TTUHSC). Thus, information such as health care discipline and campus location were not captured. The total number of complete responses analyzed was 673. These participants’ perspectives are particularly valuable, as all of those who took the survey had significant exposure to how health care systems function through their diverse experiences with TTUHSC. The TTUHSC includes the School of Medicine, School of Nursing, School of Health Professions, and School of Pharmacy spread across campuses in Lubbock, Amarillo, Dallas, El Paso, Midland, and Odessa. However, no participants outside the TTUHSC system or under the age of 18 years were included in this study. The survey measured overall social media utilization among different age groups, occupations, and education levels, along with interest in social media directed toward health care. The survey also assessed what forms of social media use would be most beneficial in facilitating the success and growth of a developing medical practice. The data collected works in conjunction with an extensive review of published literature to show the demand for social media utilization in health care, while providing a perspective from a unique population of health care faculty and students affiliated with health sciences centers in West Texas.

Respondents had 2 weeks to respond to the survey. The survey included 12 questions in a variety of formats that took 3 to 5 minutes to complete. The survey was distributed by email to an automatically generated, random list of approximately 5000 people affiliated with each TTUHSC campus. This survey was conducted by self-selection (to limit bias, respondents did not know the topic of the survey until after beginning it) and was optional, so no follow-up was carried out.

The population was chosen based on a unique and potentially valuable perspective on how social media can be implemented successfully in a health care setting from those who have had experience in the field. These data were then analyzed by age to gain insight into how opinions on social media changed based on different levels of experience in their health care careers as well as different stages of life. The value of our data is focused on providing a more focused analysis of data based on those with experience in the health care field. We hope this additional insight will provide benefits to those attempting to implement or improve social media utilization to contribute to the development of their health care practice.

### Measures and Data Collection

The survey assesses social media use in general as well as the current and prospective implications of social media use in health care across different platform options. The social media platforms assessed were Facebook, Instagram, Twitter, LinkedIn, and YouTube. Differences in use across social media platforms were measured by requiring respondents to choose from six options assessing use frequency. The options included *I do not use this platform*, *I use this platform monthly*, *I use this platform weekly*, *I use this platform daily*, *I use this platform hourly*, or *I use this platform more than once per day*. These responses were scaled from 0 to 5 and are presented in Table 1. The comfort level of respondents with different social media platforms being used in a health care setting was measured through comparison by asking respondents to rank the different social media platforms from most comfortable to least comfortable with each platform being used in health care. The responses were scaled from 1 to 7 and are presented in Table 2. In addition, a variety of subjective questions were included to better understand the amount of social media use and the preference of such use among respondents in a health care setting. Respondents were also asked about concerns that they may have with integrating social media into their health care experience. All collected responses were assessed in groups defined by age.

Responses were defined by age prospectively, using the internal TTUHSC data. Age ranges were based on stages of life or career: 18 to 25 years, students; 26 to 35 years, interns or early career; 36 to 45 years, rapid career advancement; 46 to 55 years, peak career attainment; and 56 to 65+ years, career maturity. Ages over 65 years were included as anyone with an active TTUHSC email would not yet be retired and continuing in the same career stage.

Age often helps distinguish patient populations of different health care practices (ie, pediatrics vs geriatrics). Aging is also a well-established risk factor for the development of multiple chronic diseases, including cardiovascular disease, stroke, cancer, osteoarthritis, and dementia [13]. Other variables, such as occupation and education, require nuanced social media strategies that are less advantageous than a strategy tailored to age groups. However, occupational and educational data-based social media strategies may benefit from further studies.

**Table 1:** Social media platform use (rated using a 0-5 scale, where 0 indicates “I do not use social media” and 5 indicates “I use the platform hourly or more than 12 times a day”) by age groups.

Social media platform	18-25 years, median (IQR)	26-35 years, median (IQR)	36-45 years, median (IQR)	46-55 years, median (IQR)	56-89 years, median (IQR)	*P* value^a^
Facebook	2 (2-3)	2 (2-3)	2 (2-2)	2 (2-3)	2 (1-3)	.06
Instagram	2 (1-2)	2 (1-3)	2 (0-4)	2 (0-3)	0 (0-3)	<.001
Twitter	1.5 (0-2)	0 (0-1)	0 (0-2)	0 (0-4)	0 (0-0.25)	<.001
LinkedIn	0 (0-5)	0 (0-5)	2 (0-5)	3 (0-5)	2 (0-5)	.12
YouTube	3 (2-4.25)	3 (2-4)	4 (2-4)	4 (2-4)	4 (2-5)	.61

^a^All *P* values were obtained from the independent samples Kruskal-Wallis test.

**Table 2:** Social media platforms that the participants are most comfortable using (ranked from 1-7, where 1 indicates the least comfortable using and 7 indicates the most comfortable using) in a health care setting by age groups.

Social media platform	18-25 years, median (IQR)	26-35 years, median (IQR)	36-45 years, median (IQR)	46-55 years, median (IQR)	56-89 years, median (IQR)	*P* value^a^
Facebook	6 (5-7)	6 (5-7)	7 (5-7)	6 (5-7)	6 (4-7)	.47
Instagram	5 (4-6)	5 (4-6)	5 (4-6)	5 (4-6)	4 (3-6)	<.001
Twitter	4 (3-5)	4 (3-5)	4 (3-5)	4 (2.5-5)	3.5 (2-4.75)	.02
LinkedIn	3 (3-5)	4 (3-5)	4 (3-5)	4 (3-6)	4.5 (3-6)	.009
YouTube	5 (4-6)	5 (4-6)	5 (3-6)	5 (3.5-6)	5 (3.25-6)	.02

^a^All *P* values were obtained from the independent samples Kruskal-Wallis test.

### Data Analysis

The data were summarized using descriptive statistics such as median (IQR) and frequency (percentage) as appropriate, depending on the level of measurement of the examined variables. A chi-square test was conducted to determine statistically significant differences in categorical variables across different age groups. The Kruskal-Wallis H test was conducted to determine the statistically significant differences in ordinal level variables across different age categories. The Dunn post hoc test adjusted with Bonferroni correction was performed for pairwise comparisons. As the Kruskal-Wallis H test compares mean ranks among groups on the examined variables, the mean ranks of groups that showed statistically significant differences were reported in addition to the medians and IQRs. Statistical significance was set at *P*<.05. All analyses were performed using the IBM SPSS software, version 25.

## Results

### General Study Population Results

A total of 5000 surveys were distributed, and there were a total of 811 responses. Due to some incomplete responses, the total usable responses were 13.46% (673/5000). Data show that 72.7% (489/673) of the sample population had concerns with social media use in health care due to lack of privacy or communication security, whereas only 4% (27/673) showed no concerns at all.

### Results of Categorical Variables Across Age Groups

Table 3 summarizes our findings from four of the most telling questions that were asked in our survey. The first of these research questions (Q8) was used to assess the current influence of health care professionals on social media by asking respondents whether they had ever followed a professional social media account of an independent physician or medical practice. Across all respondents, 48.4% (326/673) answered “yes,” 12.8% (86/673) answered “no, but I would like to if that was an option,” leaving only 38.8% (261/673) of respondents who had never intended to follow a health care professional. There was a statistically significant association between age groups and the above response (*χ*^2^_8_=82.6; *P*<.001; Table 3). This difference between age groups was most apparent in respondents aged 56-89 years, of which the majority (81/111, 73%) indicated that they would generally not follow a professional social media account of an independent physician or medical practice.

The next research question (Q10) was used to gauge the utility of a doctor with an updated LinkedIn account to share his or her achievements and educational or professional history. A total of 76.4% (514/673) of respondents indicated that they would find it beneficial if their physician had a public LinkedIn account. However, as in the first question, chi-square tests of the respondents’ answers were significantly different by age (*χ*²_8_=40.2; *P*<.001; Table 3).

The following question (Q11) was used to garner patient interest in following or using social media for personal medical use, such as scheduling appointments. Three responses were included, as shown in Table 3, with responses differing by the degree of interest shown in using social media for this purpose. In total, 56.3% (379/673) of respondents said that they would follow a social media page that allows them to schedule appointments and contact their nurse or doctor directly to ask questions. However, only 43.7% (294/673) of respondents preferred this over a traditional web page. As with the previous research questions, these responses also differed significantly by age (*χ*²_8_=19.8; *P*=.01). Respondents aged 56-89 years were significantly different when compared with all other ages, with 59.5% (66/111) of them indicating that they would not even follow the page (Table 3).

The final question shown in Table 3 (Q12) was used to assess the degree to which social media could be used to improve the likelihood of patients scheduling recommended screening tests. The responses, based on four selections ranging from no benefit to large improvement, showed that 46.8% (315/673) of the survey population would be more likely to schedule critical screening tests after seeing an educational social media post that provides links that would allow them to schedule an appointment. As with the other questions, however, chi-square analysis (*χ*²_8_=50.1; *P*<.001) revealed that these responses varied significantly by age. The likelihood decreased with increasing age of the sample population. Only 24.3% (27/111) of those aged over 55 years were more likely to schedule an appointment.

**Table 3:** Differences in categorical variables across categories of age groups.

Survey questions		18-25 years (n=154), n (%)	26-35 years (n=171), n (%)	36-45 years (n=133), n (%)	46-55 years (n=104), n (%)	56-89 years (n=111), n (%)	*P* value^a^	
**Q8. Have you ever followed a professional (not personal) social media account of an independent physician or medical practice?**								<.001
	Yes	77 (50)	98 (57.3)	76 (57.1)	52 (50)	23 (20.7)		
	No	43 (27.9)	52 (30.4)	41 (30.8)	44 (42.3)	81 (73)		
	No, but I would like to if that was an option	34 (22.1)	21 (12.3)	16 (12)	8 (7.7)	7 (6.3)		
**Q10. Would you find it beneficial from a patient’s perspective for your doctor to have an updated, public LinkedIn account that would allow you to have more access to his or her professional history, achievements, and education?**								<.001
	Yes, this would help me develop confidence in my physician and add credibility to the guidance he or she gives me	71 (46.1)	86 (50.3)	62 (46.6)	44 (42.3)	44 (39.6)		
	Yes, but probably would not check it anyway	68 (44.2)	46 (26.9)	40 (30.1)	28 (26.9)	25 (22.5)		
	No, I do not think that would be useful or beneficial	15 (9.7)	39 (22.8)	31 (23.3)	32 (30.8)	42 (37.8)		
**Q11. As a patient would you be inclined to follow and use a social media page (Instagram, Facebook, etc) to contact your nurse or doctor directly to get medical questions answered, schedule appointments, and get updates? Would this be more convenient than using a conventional web page?**								.01
	Absolutely, this would be convenient	58 (37.7)	62 (36.3)	52 (39.1)	34 (32.7)	27 (24.3)		
	I would follow the social media account but probably never take advantage	40 (25.9)	42 (24.5)	22 (16.5)	24 (23.1)	18 (16.2)		
	I would not be interested in the social media account and would just use a regular website for the information I need	56 (36.3)	67 (39.2)	59 (44.4)	46 (44.2)	66 (59.5)		
**Q12. Would you be more likely to schedule critical screening tests such as mammograms or colonoscopies if you saw an educational post on social media explaining the importance of them and providing a convenient link that would allow you to directly schedule an appointment?**								<.001
	Yes, this would help me remember to get important preventive testing	86 (55.9)	90 (52.6)	64 (48.1)	48 (46.2)	27 (24.3)		
	This would be beneficial and educational, but I probably would not be inclined to schedule an appointment through the post	51 (33.1)	49 (28.7)	36 (27.1)	33 (31.7)	37 (33.3)		
	If I saw the post, I would not pay much attention to it	10 (6.5)	16 (9.4)	16 (12)	4 (3.8)	17 (15.3)		
	This would not benefit me	7 (4.5)	16 (9.4)	17 (12.8)	19 (18.3)	30 (27)		

^a^All the *P* values are obtained from the Pearson chi-square test.

### Social Media Use by Age

The Kruskal-Wallis test was conducted to determine the differences in social media platform use (rated using a 0-5 scale, where 0 indicates *I do not use social media* and 5 indicates *I use the platform hourly more than 12 times a day*) across different age groups. Statistically significant differences were found among different-aged Instagram users (*P*<.001) and Twitter users (*P*<.001; Table 1). Post hoc tests for use of Instagram revealed that the use differed significantly between the age groups 56-89 years (median 0, IQR 0-3) and 18-25 years (median 2, IQR 1-2; mean ranks, respectively, 217-316; *P*=.03), 56-89 years (median 0, IQR 0-3) and 26-35 years (median 2, IQR 1-3; mean ranks, respectively, 217-313; *P*<.001), 56-89 years (median 0, IQR 0-3) and 36-45 years (median 2, IQR 0-4; mean ranks, respectively, 217-311; *P*=.001), and 56-89 years (median 0, IQR 0-3) and 46-55 years (median 2, IQR 0-3; mean ranks, respectively, 217-314; *P*=.02), but the use did not differ between any other age group combination. As for the use of Twitter, the post hoc test showed that there was a significant difference between age groups 56-89 years (median 0, IQR 0-2.5) and 18-25 years (median 1.5, IQR 0-2; mean ranks, respectively, 255-319; *P*=.16), 56-89 years (median 0, IQR 0-2.5) and 46-55 years (median 0, IQR 0-4; mean ranks, respectively, 255-322; *P*=.03), 26-35 years (median 0, IQR 0-1) and 18-25 years (median 1.5, IQR 0-2; mean ranks, respectively, 257-318; *P*=.002), 26-35 years (median 0, IQR 0-1) and 46-55 years (median 0, IQR 0-4; mean ranks, respectively, 257-322; *P*=.009), and 36-45 years (median 0, IQR 0-2) and 18-25 years (median 1.5, IQR 0-2; mean ranks, respectively, 258-319; *P*=.04), but the use did not differ between any other age group combination.

Of the survey population, 76.8% (517/673) claimed to follow a form of social media that regularly posts something educational related to the medical field. Facebook was the most frequently used social media platform and was considered most acceptable for use in a health care setting across all ages surveyed. A total of 58.8% (396/673) of the sample population checked Facebook multiple times a day, and the use varied with each social media platform (Multimedia Appendix 1).

### Social Media Comfort in Health Care by Age

The Kruskal-Wallis test was also conducted to determine the significant differences in social media platforms that the participants are most comfortable using (ranked from 1-7, where 1 indicates the least comfortable using and 7 indicates the most comfortable using) in a health care setting that differed by age groups. Across various categories of age, except for *Facebook*, the participants’ responses varied significantly by age group for Instagram (*P*<.001), Twitter (*P*=.02), LinkedIn (*P*=.009), and YouTube (*P*=.02) in a health care setting (Table 2). A post hoc test showed that there was a statistically significant difference in Instagram use in a health care setting between age groups 56-89 years (median 4, IQR 3-6) and 18-25 years (median 5, IQR 4-6; mean ranks, respectively, 253-346; *P*=.001) and between age groups 46-55 years (median 5, IQR 4-6) and 18-25 years (median 5, IQR 4-6; mean ranks, respectively, 270-346; *P*=.008), but the use did not differ between any other age group combination. As for comfort using Twitter, the post hoc analysis revealed a statistically significant difference between age groups 56-89 years (median 3.5, IQR 2-4.7) and 18-25 years (median 4, IQR 3-5; mean ranks, respectively, 259-332; *P*=.02), but the use did not differ between any other age group combination. For LinkedIn, there was a statistically significant difference between age groups 56-89 years (median 4.5, IQR 3-6) and 18-25 years (median 3, IQR 3-5; mean ranks, respectively, 334-261; *P*=.02) and age groups 46-55 years (median 4, IQR 3-6) and 18-25 years (median 3, IQR 3-5; mean ranks, respectively, 325-260; *P*=.04), but the use did not differ between any other age group combination. Finally, for YouTube, the post hoc test revealed a statistically significant difference between age groups 36-45 years (median 5, IQR 3-6) and 26-35 years (median 5, IQR 4-6; mean ranks, respectively, 262-333; *P*=.006), but the use did not differ between any other age group combination.

## Discussion

### Principal Findings

The growing interest and influence of social media in the general public undoubtedly poses the following question [1]: why is this not being more heavily used in health care? The current explanation is that the apprehensions toward social media stem from quality, reliability, confidentiality, and privacy concerns [7]. More specifically, the most common contributors to individual and institutional fear against the use of social media in medicine and health care may include the potential violation of ethical standards, patient privacy, confidentiality, and the misrepresentation of information. According to our survey, the greatest concerns were lack of privacy (258/673, 38.3%) and communication security (231/673, 34.3%). Despite these concerns, a strong social media presence can be used to fortify a positive reputation as a medical practice. It can also be an effective way to educate the followers on important medical topics, which in turn could lead to further patient acquisition. Along with educating patients, another possible improvement to a developing medical practice is increased patient satisfaction through possibilities such as improved patient adherence [14]. The possible implications of efficient utilization of social media will continue to grow over time, but many developing medical practices that have not yet started to take advantage of these opportunities are possibly missing out on significant improvements in several areas.

Despite the concerns expressed with social media use in the medical field, the vast majority of respondents showed strong interest in greater social media involvement in health care. The results were relatively consistent between respondents aged 18-55 years, but those aged over 55 years appear to express a change in outlook on social media involvement in health care. The majority of the data’s significant findings were from the abrupt change in the opinion of the older respondents. The trend showed a steady decrease in the interest of social media utilization in health care, as each age group increased until a steep drop was found after 55 years of age (Table 3). For example, about 46.8% (315/673) of respondents indicated that they would be more likely to schedule critical screening tests after seeing an educational social media post that provides a link that would allow them to schedule an appointment. However, the likelihood decreased with increasing age of the sample population, and less than 24.3% (27/111) of those aged over 55 years were more likely to schedule an appointment (Table 3; *P*<.001). Another finding separating the opinion of those aged over 55 years was when asked if respondents followed a professional (not personal) social media account of an independent physician or medical practice. About 48.4% (326/673) of the respondents indicated that they did. However, when analyzed by age, the majority (81/111, 73.2%) of respondents aged 56-89 years indicated that they would not follow a professional social media account of an independent physician or medical practice (Table 3; *P*<.001). Finally, 56.3% (379/673) of respondents indicated that they would follow a social media page that allows them to schedule appointments and contact their nurse or doctor directly to ask a question. However, only 43.7% (294/673) of respondents would prefer this over a traditional web page, and respondents aged 56-89 years were significantly different from the other groups, with 59.5% (66/111) indicating that they would not even follow the page (*P*=.009; Table 3). The majority of respondents within all age groups expressed that it would be beneficial from a patient’s perspective to have a doctor with a public, updated LinkedIn account, allowing more details on their professional history. However, this was expressed more conclusively among younger respondents aged between 18 and 25 years (71/154, 46.1%) than among older respondents aged between 56 and 89 years (44/111, 39.6%; Table 3).

It could be valuable to consider how often each platform is being checked and by what demographic. Although it is likely that health care providers are more prone to follow social media regarding education in health care, these data still provide value because they show that the majority of health care professionals of all ages (the survey population had a relatively even distribution of ages) find value in social media. The data also allow us to further analyze which social media platforms are preferred for medical-related content by health care professionals of different age groups.

Facebook and Instagram are the platforms most often checked multiple times a day, where YouTube appears to be a weekly habit and LinkedIn monthly. The majority of respondents did not use Twitter, but those that used Twitter checked it frequently (Multimedia Appendix 1). The survey data measured which social media platforms could be most successful in a health care setting by comparing differences in use and comfort in a health care setting among different age groups. Facebook was the most frequently used social media platform and was considered most acceptable for use in a health care setting across all ages (Multimedia Appendix 1). However, statistically significant differences in age groups were found between respondents’ use of both Instagram and Twitter. There were no significant differences between the 18 to 55 years age group, but the 56 to 89 years age group used Instagram significantly less than each of the other age groups (Table 1). The 56-89 years age group recorded a median of 0, meaning no use at all, whereas all other age groups reported significantly different use. The 18 to 25 years (*P*=.03), 26 to 35 years (*P*<.001), 36 to 45 years (*P*=.001), and 46 to 55 years (*P*=.02) age groups all recorded a median of 2, indicating almost daily use. Twitter also showed a similar variation in use by age. The 56 to 89 years age group reported infrequent to no use of Twitter at all, with a median of 0, which was significantly less than the 18 to 25 years age group that reported monthly to weekly use (median 1.5; *P*=.16). Twitter showed that the 46 to 55 years age group also differed significantly, with more frequent use than the 56 to 89 years age group (*P*=.03). However, the 46 to 55 years age group recorded a significantly less frequent use of Twitter when compared with the 26 to 35 years age group (*P*=.009; Table 1). Clearly, certain social media platforms such as Instagram and Twitter are more favorably adopted among younger populations. Understanding these differences could be vital to the implementation of successful and efficient strategies to use social media in a developing health care practice.

Considering the reservations to increased social media in the medical field that have been expressed, understanding the different levels of comfort for each social media platform in a health care setting could have a significant impact on the success of social media utilization. Levels of comfort among different social media platforms showed similar significant differences between age groups. The 56 to 89 years age group expressed significantly less comfort with the utilization of Instagram in health care (median 4) when compared with the 18 to 25 years age group (median 5; *P*=.001). The 46 to 55 years age group also recorded less comfort with Instagram in health care when compared with the 18 to 25 years age group (*P*=.008; Table 2). It is important to consider this decrease in comfort with increasing age for any social media utilization plan involving Instagram in health care. Twitter also showed a significant difference in comfort level using the platform in a health care setting when comparing the 56 to 89 years age group with the 18 to 25 years age group. The older populations (aged 56-89 years) showed significantly less comfort with Twitter’s use in health care (median 3.5) compared with those aged 18 to 25 years (median 4; *P*=.02; Table 2). As such, these platforms may be less useful for physicians in geriatric care than those in specialties with younger patients. For example, pediatric practices may benefit from these platforms, as the appointments are generally scheduled by parents that may fall in surveyed ages between 18 and 46 years. Interestingly, not all social media platforms showed decreased comfort with utilization in the health care setting in the older age groups. LinkedIn actually followed the opposite trend. The 56 to 89 years age group showed significantly more comfort with LinkedIn utilization in the health care setting (median 4.5) when compared with the 18 to 25 years age group (median 3; *P*=.02). The 46 to 55 years age group also showed significantly more comfort with LinkedIn in a health care setting (median 4) when compared with the 18 to 25 years age group (median 3; *P*=.04). These data show that LinkedIn could be a valuable tool for a medical practice wanting to appeal to an older patient population when implementing a social media utilization plan.

With these data in mind, it is reasonable to conclude that younger respondents tend to be more active and comfortable on social media, so the platforms they most commonly use will be checked on a more frequent basis. This should be considered when targeting specific demographics for educational videos or patient acquisition. For example, two platforms that were not included in the survey data that serve younger demographics are Snapchat and TikTok. Snapchat is most frequently used by people aged between 13 and 29 years, with 69% of 13- to 17-year-olds using the app and 62% of 18- to 29-year-olds using the app. Snapchat reached 210 million daily users in the fourth quarter of 2019. For this reason, Snapchat may not be the best option for health care–related use and was not included in the survey, but it would be worth considering in the future if it retains its current user base. The platform TikTok gained significant popularity after beginning this research and was not included in the survey data. However, it has since become a major platform with rapid growth and could be a strong tool in a future health care social media program. Although more data would need to be collected on its effectiveness in the health care setting, TikTok may be a strong option because its 800 million active users spend an average of 52 minutes per day on the app worldwide. Only 41% of the users were aged between 16 and 24 years, so there are many over the age of 25 years. The higher the active user base, the more likely a health care practice will be able to reach or target specific patient populations. Different social media platforms may be used in different ways to accomplish their objectives, but the intrinsic value of social media is the ability to reach a larger and diverse audience.

If a health care organization was trying to improve patient acquisition or reach a broader audience, the survey data suggest that optimization of social media programs requires consideration of patient demographics, especially targeting the platform type and time and use of each platform based on age. The styles of social media utilization with the first and second most interest among survey respondents were posts that address important medical topics each month with short weekly educational videos from a physician specializing in that particular area and live social media question and answer sessions, respectively. Despite the overwhelming amount of data suggesting that social media could be an excellent resource in the health care industry, some data indicate that there are significant concerns that may prevent efficient adoption. The majority of respondents across all age groups reported that they would not take advantage of a social media page that facilitated direct communication to receive answers to medical questions, schedule appointments, or receive general updates (Table 3). Further research is needed to better understand the possible impact of the concerns related to privacy and security of communication on the ease of general patient adoption of social media in the health care industry. Those interested in more generalizable demographics could repeat this survey with a larger sample population, including people with occupations in a variety of industries across various geographic locations in the United States. This could provide valuable insights into the most effective social media utilization in health care for different target populations. Although the future applications and growth in popularity of patients using social media to seek out medical guidance are currently unknown, the data from this survey and other available data suggest that social media utilization has room to grow and may play a more prominent role in health care. The younger generations who spend significant amounts of time each day on social media will eventually be responsible for the majority of health care spending, which could allow social media to be a powerful tool for many medical practices in the future.

### Limitations and Future Research

This study included participants with higher education and experience in the Texas health care industry; however, this presents limitations due to the lack of geographical location and occupational diversity among all respondents. Further studies would benefit from including more respondents who are not affiliated with the health care system and respondents from a broader geographical distribution to improve generalizability and further understand how the public would react to increased social media utilization in the medical field. Furthermore, the survey was optional and was sent to anyone with a ttuhsc.edu email. Although this enabled a large sample size, this study design allows for self-selection, which may create a bias in the responses.

Although some challenges of health care utilization have already been identified, it would be helpful to expand on these challenges in further studies, especially addressing misinformation spread through social media in the health care field. In addition, an attempt to understand the higher use preference of LinkedIn by older age groups could help shed more light on this reverse trend compared with other social media platforms, and we recommend this as an area of future study.

### Conclusions

As social media continues to grow, efficient utilization of the available platforms can help a medical practice reach out to a broader population and deliver personalized care. Although the data collected in this study demonstrated an overwhelming interest in using social media in the medical field across all age groups, adoption willingness appears to be higher in younger respondents than in older respondents. Facebook is the most widely accepted social media platform for health care applications. However, other social media platforms, such as Instagram, may be better tools for targeting younger generations. Medical practices should use social media pages to present content that is timely, relevant, and written in a clear language familiar to the target audience.

Furthermore, physicians are encouraged to have updated LinkedIn profiles to gain the attention of more potential patients and to increase patients’ confidence in their physicians. Respondents aged over 55 years seem to be less receptive to following health care–related social media pages and are particularly less receptive to using social media over a traditional web page. However, based on the majority of survey responses, there is great interest in the availability of educational health care videos on social media, access to health care providers, and appointment scheduling via hyperlinks. It is plausible that using social media in these ways could lead a medical practice to an increase in patient acquisition and improved health care delivery. There are significant concerns related to information accuracy, privacy, and security that need to be addressed to improve outcomes from social media use in the medical field. However, the current benefits and future possibilities of social media utilization make it a powerful and strategic option for medical practices to adopt.

### Recommendations

**Figure 1:**
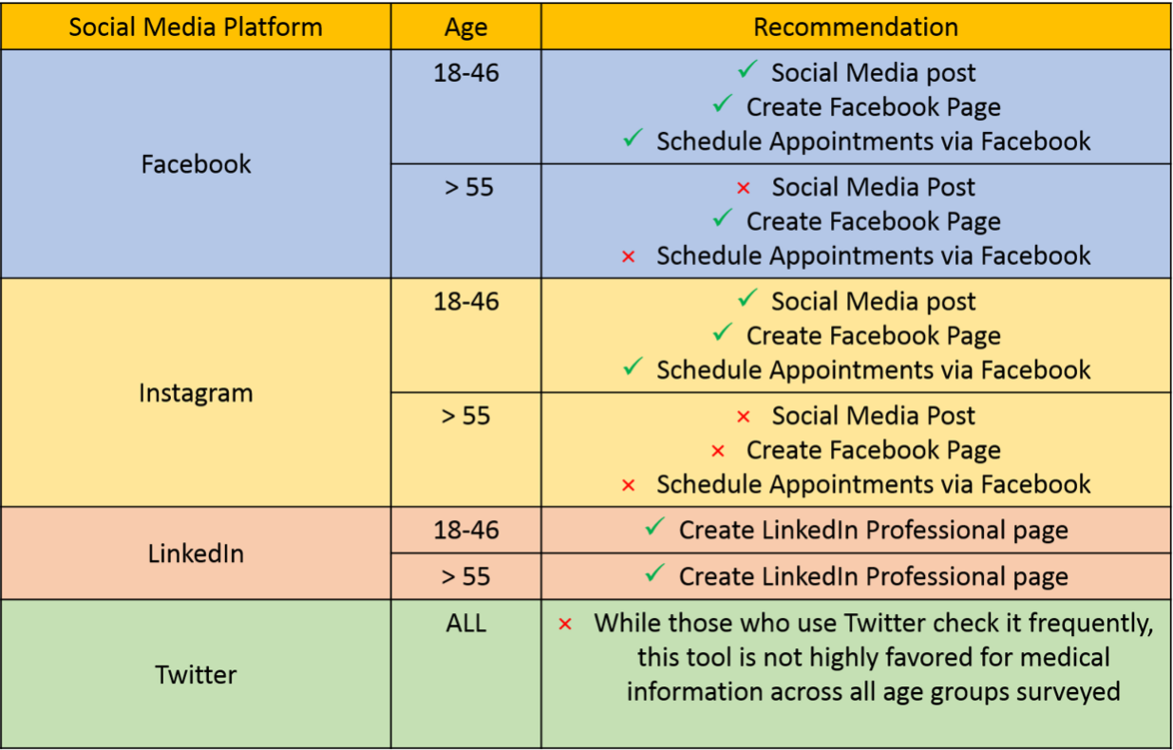
Recommendations for social media strategies in health care based on age.

On the basis of our data, we recommend that all physicians have an updated LinkedIn account, which could improve the patient-physician relationship as well as ensure patients’ confidence in their physician, among all patients aged over 18 years (Table 3; Figure 1).

Growing medical practices that are implementing a social media utilization plan should focus on patient age when targeting different patient populations. Stratifying by patient age showed more significant associations in our data and is likely more accessible information than factors such as occupation and education when implementing a social media outreach plan in a health care setting. As all age groups were more comfortable with Facebook in a health care setting and checked Facebook most frequently **(**Multimedia Appendix 1; Figure 1), it would likely be the most effective platform when targeting patient populations with a broad age range (18-89 years). Facebook supplemented with LinkedIn could be more effective when targeting patient populations aged over 46 years. Instagram along with Facebook could be effective in targeting patients aged under 46 years. As most medical practices have patients of all ages, our research supports a multifaceted approach that includes multiple social media platforms uniquely used to target different age groups (Figure 1).

## Appendix

Multimedia Appendix 1 Current usage of social media among different age groups.

## Abbreviations

TTUHSC: Texas Tech University Health Sciences Center

## References

[1] Demographics of social media users and adoption in the United States. Pew Research Center.

[2] Antheunis ML, Tates K, Nieboer TE (2013). Patients' and health professionals' use of social media in health care: motives, barriers and expectations. Patient Educ Couns.

[3] Kohli MD, Daye D, Towbin AJ, Kotsenas AL, Heilbrun ME (2018). Social media tools for department and practice communication and branding in the digital age. Radiographics.

[4] Grajales FJ, Sheps S, Ho K, Novak-Lauscher H, Eysenbach G (2014). Social media: a review and tutorial of applications in medicine and health care. J Med Internet Res.

[5] Santoro E (2013). [Social media and medical apps: how they can change health communication, education and care]. Recenti Prog Med.

[6] Martin A, Grundin E, Harrison D, Espinoza J (2012). Marketing your practice in a social world. J Med Pract Manage.

[7] Moorhead SA, Hazlett DE, Harrison L, Carroll JK, Irwin A, Hoving C (2013). A new dimension of health care: systematic review of the uses, benefits, and limitations of social media for health communication. J Med Internet Res.

[8] Hawkins CM, DeLa OJ, Hung C (2016). Social media and the patient experience. J Am Coll Radiol.

[9] Barreto JE, Whitehair CL (2017). Social media and web presence for patients and professionals: evolving trends and implications for practice. PM R.

[10] Denecke K, Bamidis P, Bond C, Gabarron E, Househ M, Lau AY, Mayer MA, Merolli M, Hansen M (2015). Ethical issues of social media usage in healthcare. Yearb Med Inform.

[11] Terrasse M, Gorin M, Sisti D (2019). Social media, e-health, and medical ethics. Hastings Cent Rep.

[12] Benetoli A, Chen T, Aslani P (2018). How patients' use of social media impacts their interactions with healthcare professionals. Patient Educ Couns.

[13] Atella V, Mortari A, Kopinska J, Belotti F, Lapi F, Cricelli C, Fontana L (2019). Trends in age-related disease burden and healthcare utilization. Aging Cell.

[14] Gijsen V, Maddux M, Lavertu A, Gonzalez-Hernandez G, Ram N, Reeves B, Robinson T, Ziesenitz V, Shakhnovich V, Altman R (2020). #Science: The potential and the challenges of utilizing social media and other electronic communication platforms in health care. Clin Transl Sci.

