# Supplementary material for: Implications of Age on Social Media Utilization in Health Care Practice Development: Cross-sectional Survey Study
Source: JMIR Hum Factors. 2021 Jul 15;8(3):e27528. doi: 10.2196/27528 (PMC8323016; doi:10.2196/27528)
Supplement: Multimedia Appendix 1 [file humanfactors_v8i3e27528_app1.docx]

**Multimedia Appendix 1.** Current usage of social media among different age groups.

| Current use of social media among different age groups - Facebook | | | | | | |  |  |  |  |  | |  |  |
| --- | --- | --- | --- | --- | --- | --- | --- | --- | --- | --- | --- | --- | --- | --- |
| # | Question | Hourly (more than 12 times a day) | | Multiple times a day | | Daily (once a day) | | Weekly (1-6 times a week) | | Monthly (3-4 times a month) | | I do not use this social media platform | | |
| 1 | 18-25 | 27.08% | 13 | 26.15% | 91 | 25.24% | 26 | 19.61% | 10 | 9.68% | 3 | | 14.29% | 10 |
| 2 | 26-35 | 35.42% | 17 | 26.15% | 91 | 21.36% | 22 | 27.45% | 14 | 29.03% | 9 | | 22.86% | 16 |
| 3 | 36-45 | 16.67% | 8 | 23.28% | 81 | 11.65% | 12 | 15.69% | 8 | 19.35% | 6 | | 21.43% | 15 |
| 4 | 46-55 | 6.25% | 3 | 15.23% | 53 | 21.36% | 22 | 21.57% | 11 | 16.13% | 5 | | 12.86% | 9 |
| 5 | 56-89 | 14.58% | 7 | 9.20% | 32 | 20.39% | 21 | 15.69% | 8 | 25.81% | 8 | | 28.57% | 20 |
|  | Total | Total | 48 | Total | 348 | Total | 103 | Total | 51 | Total | 31 | | Total | 70 |
|  |  |  |  |  |  |  |  |  |  |  |  | |  |  |
| Current use of social media among different age groups - Instagram | | | | | | |  |  |  |  |  | |  |  |
| # | Question | Hourly (more than 12 times a day) | | Multiple times a day | | Daily (once a day) | | Weekly (1-6 times a week) | | Monthly (3-4 times a month) | | I do not use this social media platform | | |
| 1 | 18-25 | 53.19% | 25 | 42.79% | 86 | 19.12% | 13 | 8.47% | 5 | 12.50% | 7 | | 9.36% | 16 |
| 2 | 26-35 | 34.04% | 16 | 30.85% | 62 | 19.12% | 13 | 35.59% | 21 | 23.21% | 13 | | 21.05% | 36 |
| 3 | 36-45 | 8.51% | 4 | 16.92% | 34 | 20.59% | 14 | 23.73% | 14 | 28.57% | 16 | | 22.22% | 38 |
| 4 | 46-55 | 2.13% | 1 | 7.96% | 16 | 30.88% | 21 | 22.03% | 13 | 19.64% | 11 | | 18.13% | 31 |
| 5 | 56-89 | 2.13% | 1 | 1.49% | 3 | 10.29% | 7 | 10.17% | 6 | 16.07% | 9 | | 29.24% | 50 |
|  | Total | Total | 47 | Total | 201 | Total | 68 | Total | 59 | Total | 56 | | Total | 171 |
|  |  |  |  |  |  |  |  |  |  |  |  | |  |  |
| Current use of social media among different age groups - Twitter | | | | | | |  |  |  |  |  | |  |  |
| # | Question | Hourly (more than 12 times a day) | | Multiple times a day | | Daily (once a day) | | Weekly (1-6 times a week) | | Monthly (3-4 times a month) | | I do not use this social media platform | | |
| 1 | 18-25 | 41.18% | 7 | 59.74% | 46 | 25.81% | 8 | 15.56% | 7 | 22.45% | 11 | | 18.64% | 66 |
| 2 | 26-35 | 29.41% | 5 | 12.99% | 10 | 16.13% | 5 | 20.00% | 9 | 24.49% | 12 | | 30.23% | 107 |
| 3 | 36-45 | 17.65% | 3 | 12.99% | 10 | 22.58% | 7 | 20.00% | 9 | 16.33% | 8 | | 22.03% | 78 |
| 4 | 46-55 | 5.88% | 1 | 12.99% | 10 | 22.58% | 7 | 31.11% | 14 | 20.41% | 10 | | 12.43% | 44 |
| 5 | 56-89 | 5.88% | 1 | 1.30% | 1 | 12.90% | 4 | 13.33% | 6 | 16.33% | 8 | | 16.67% | 59 |
|  | Total | Total | 17 | Total | 77 | Total | 31 | Total | 45 | Total | 49 | | Total | 354 |
|  |  |  |  |  |  |  |  |  |  |  |  | |  |  |
| Current use of social media among different age groups - LinkedIn | | | | | | |  |  |  |  |  | |  |  |
| # | Question | Hourly (more than 12 times a day) | | Multiple times a day | | Daily (once a day) | | Weekly (1-6 times a week) | | Monthly (3-4 times a month) | | I do not use this social media platform | | |
| 1 | 18-25 | 0.00% | 0 | 5.00% | 1 | 14.29% | 4 | 14.49% | 10 | 23.60% | 38 | | 28.57% | 84 |
| 2 | 26-35 | 33.33% | 1 | 10.00% | 2 | 21.43% | 6 | 28.99% | 20 | 24.22% | 39 | | 28.57% | 84 |
| 3 | 36-45 | 33.33% | 1 | 30.00% | 6 | 28.57% | 8 | 15.94% | 11 | 22.36% | 36 | | 17.69% | 52 |
| 4 | 46-55 | 0.00% | 0 | 25.00% | 5 | 25.00% | 7 | 21.74% | 15 | 15.53% | 25 | | 11.22% | 33 |
| 5 | 56-89 | 33.33% | 1 | 30.00% | 6 | 10.71% | 3 | 18.84% | 13 | 14.29% | 23 | | 13.95% | 41 |
|  | Total | Total | 3 | Total | 20 | Total | 28 | Total | 69 | Total | 161 | | Total | 294 |
|  |  |  |  |  |  |  |  |  |  |  |  | |  |  |
| Current use of social media among different age groups - You Tube | | | | | | |  |  |  |  |  | |  |  |
| # | Question | Hourly (more than 12 times a day) | | Multiple times a day | | Daily (once a day) | | Weekly (1-6 times a week) | | Monthly (3-4 times a month) | | I do not use this social media platform | | |
| 1 | 18-25 | 35.29% | 6 | 30.97% | 35 | 29.27% | 24 | 17.24% | 30 | 25.90% | 36 | | 17.07% | 14 |
| 2 | 26-35 | 47.06% | 8 | 32.74% | 37 | 26.83% | 22 | 27.01% | 47 | 21.58% | 30 | | 17.07% | 14 |
| 3 | 36-45 | 5.88% | 1 | 15.93% | 18 | 14.63% | 12 | 24.71% | 43 | 16.55% | 23 | | 31.71% | 26 |
| 4 | 46-55 | 0.00% | 0 | 14.16% | 16 | 17.07% | 14 | 18.97% | 33 | 13.67% | 19 | | 13.41% | 11 |
| 5 | 56-89 | 11.76% | 2 | 6.19% | 7 | 12.20% | 10 | 12.07% | 21 | 22.30% | 31 | | 20.73% | 17 |
|  | Total | Total | 17 | Total | 113 | Total | 82 | Total | 174 | Total | 139 | | Total | 82 |

Table 4 – Current usage of social media among different age groups
